# Proposed guidelines to evaluate scientific validity and evidence for genotype-based dietary advice

**DOI:** 10.1186/s12263-017-0584-0

**Published:** 2017-12-15

**Authors:** Keith A. Grimaldi, Ben van Ommen, Jose M. Ordovas, Laurence D. Parnell, John C. Mathers, Igor Bendik, Lorraine Brennan, Carlos Celis-Morales, Elisa Cirillo, Hannelore Daniel, Brenda de Kok, Ahmed El-Sohemy, Susan J. Fairweather-Tait, Rosalind Fallaize, Michael Fenech, Lynnette R. Ferguson, Eileen R. Gibney, Mike Gibney, Ingrid M. F. Gjelstad, Jim Kaput, Anette S. Karlsen, Silvia Kolossa, Julie Lovegrove, Anna L. Macready, Cyril F. M. Marsaux, J. Alfredo Martinez, Fermin Milagro, Santiago Navas-Carretero, Helen M. Roche, Wim H. M. Saris, Iwona Traczyk, Henk van Kranen, Lars Verschuren, Fabio Virgili, Peter Weber, Jildau Bouwman

**Affiliations:** 1 Eurogenetica Ltd, 7 Salisbury Road, Burnham-on-Sea, TA8 1HX UK; 2 TNO, Utrechtseweg 48, Zeist, The Netherlands; 3 JMUSDA-Human Nutrition Research Center on Aging at Tufts University, Boston, USA; 4 IMDEA Alimentacion, Madrid, Spain; 5 Agriculture Research Service, USDA, Human Nutrition Research Center on Aging, Boston, MA 02111 USA; 6 Human Nutrition Research Centre, Institute of Cellular Medicine, Campus for Ageing and Vitality, Newcastle University, Newcastle upon Tyne, NE4 5PL UK; 7 DSM Nutritional Products, Kaiseraugst, Switzerland; 8 UCD Institute of Food and Health, UCD School of Agriculture and Food Science, University College Dublin, Dublin, Republic of Ireland; 9 BHF Glasgow Cardiovascular Research Centre, Institute of Cardiovascular and Medical Science, University of Glasgow, Glasgow, G12 8TA UK; 10 Nutritional Physiology, Technische Universität München, 85350 Freising, Germany; 11 Department of Nutritional Sciences, University of Toronto, 150 College Street, 3rd Floor, Toronto, ON M5S 3E2 Canada; 12 Norwich Medical School, University of East Anglia, Norwich, NR4 7UQ UK; 13 Hugh Sinclair Unit of Human Nutrition and Institute for Cardiovascular and Metabolic Research, Department of Food and Nutritional Sciences, University of Reading, Whiteknights, PO Box 226, Reading, Berkshire RG6 6AP UK; 14 CSIRO Health and Biosecurity, Gate 13, Kintore Avenue, Adelaide, SA 5000 Australia; 15 ACSRC and Discipline of Nutrition and Dietetics, Faculty of Medical and Health Sciences, University of Auckland, Private Bag 92019, Auckland, 1184 New Zealand; 16 Department of Nutrition, Universitetet i Oslo, PO Box 1046, Blindern, N-0316 Oslo, Norway; 17 Vydiant Inc, 2330 Gold Meadow Way, Gold River, 95670 CA USA; 18 Department of Human Biology, NUTRIM School of Nutrition and Translational Research in Metabolism, Maastricht University Medical Centre + (MUMC+), Maastricht, The Netherlands; 19 Department of Nutrition, Food Science and Physiology, Centre for Nutrition Research, University of Navarra, Pamplona, Spain; 20 CIBERobn, Fisiopatología de la Obesidad y Nutrición, Instituto de Salud Carlos III, Madrid, Spain; 21 Nutrigenomics Research Group, UCD Institute of Food and Health/UCD Conway Institute, University College Dublin, Dublin, Ireland; 22 Department of Human Nutrition, Faculty on Health Sciences, Medical University of Warsaw, Warsaw, Poland; 23 Institute for Public Health Genomics (IPHG), Department of Genetics and Cell Biology, Faculty of Health, Medicine & Life Sciences, University of Maastricht, Universiteitssingel 40, 6229 ER Maastricht, The Netherlands; 24 Council for Agricultural Research and Economics, Food and Nutrition Research Centre, (CREA - AN), via Ardeatina 546, 00178 Rome, Italy

**Keywords:** Genotype, Dietary advice, Gene-environment interaction, Nutrigenetics, Personalised nutrition, Framework, Genetic variants, Health

## Abstract

Nutrigenetic research examines the effects of inter-individual differences in genotype on responses to nutrients and other food components, in the context of health and of nutrient requirements. A practical application of nutrigenetics is the use of personal genetic information to guide recommendations for dietary choices that are more efficacious at the individual or genetic subgroup level relative to generic dietary advice. Nutrigenetics is unregulated, with no defined standards, beyond some commercially adopted codes of practice. Only a few official nutrition-related professional bodies have embraced the subject, and, consequently, there is a lack of educational resources or guidance for implementation of the outcomes of nutrigenetic research. To avoid misuse and to protect the public, personalised nutrigenetic advice and information should be based on clear evidence of validity grounded in a careful and defensible interpretation of outcomes from nutrigenetic research studies. Evidence requirements are clearly stated and assessed within the context of state-of-the-art ‘evidence-based nutrition’. We have developed and present here a draft framework that can be used to assess the strength of the evidence for scientific validity of nutrigenetic knowledge and whether ‘actionable’. In addition, we propose that this framework be used as the basis for developing transparent and scientifically sound advice to the public based on nutrigenetic tests. We feel that although this area is still in its infancy, minimal guidelines are required. Though these guidelines are based on semi-quantitative data, they should stimulate debate on their utility. This framework will be revised biennially, as knowledge on the subject increases.

## Background

Personalised nutrition (PN), based on an individual’s genotype, is not new. Individuals with rare genetic disorders such as phenylketonuria, galactosemia and hereditary fructose intolerance [1–3]) as well as more common disorders (e.g. lactose intolerance, coeliac disease [4, 5]) tailor food intake to bypass the metabolic deficiency. In each case, specific dietary recommendations have been defined, validated and are in use clinically. A key question for the clinical and nutrition community is whether matching nutrient intake and physical activity to individual gene variants, that usually produce weaker metabolic effects compared to those mentioned above, would have noticeable impacts on health status.

Many sectors of modern society increasingly focus on personalising services or products to enable individuals to control more aspects of their lives. Public interest in personalised health has spurred growth in nutrigenetic testing services not only for disease susceptibilities but, especially, for optimising nutrition. Genetic testing is a largely unregulated market with many unsupported claims and inadequate explanations of the results and implications [6–11]. It is important to be aware that the effects of common variants will be mostly small in the overall phenotype and which will depend on several variants, gene-gene and gene-protein interactions. A common weakness in the commercial area (for all of nutrition) is to provide simple “answers” to a complex question. Hence, the aim of this paper is to provide a draft framework for assessing the validity of genetic information for the development of precise personalised dietary advice that does not go beyond the evidence.

The general objective of PN is to maintain or improve health by using genetic, phenotypic, clinical, dietary and other information to provide more precise and more efficacious personalised healthy eating advice and to motivate appropriate dietary changes. For example, about 15 years ago, it was proposed that:

“With the identification of polymorphisms, or common mutations, in vitamin metabolism, large percentages of the population may have higher requirements for specific vitamins ([12]).”

Research since then has resulted in significant progress and has identified some well-defined gene × diet interactions supporting the concept that diets tailored to the individual’s genotype might result in long-term health benefits [9, 13, 14]. Personalised advice should be more precise and more efficacious than generic advice. To facilitate acceptance by the public and other stakeholders, it is important that the development of genotype-dependent advice is based on sound evidence. The debate about direct to consumer (DTC) sales of genetic testing and the regulation of this market ([10, 15–18]), are beyond the scope of this article. DTC is a reality that makes it important that information about links between genetic variants, nutrition, and health and reliable interpretation of that information are accessible to all stakeholders including healthcare professionals, regulators, companies and the public.

The development of the framework described herein is part of a larger project within the EU FP7 project Food4Me (www.food4me.org), which was initiated to examine several aspects of PN including business models, market readiness, ethics and public perceptions, and to perform a Europe-wide proof of principle intervention study on the effectiveness of PN approaches [19, 20]. A specific goal within the Food4Me project was the creation of a publicly available resource listing gene × diet interactions [21]. PN may contribute to improved eating patterns and help reduce the burden of many common health problems including obesity, age-related diseases such as type 2 diabetes (T2D), cardiovascular disease (CVD), dementia, musculoskeletal problems and some cancers [22]. Therefore, when validated, scientific evidence of potential benefit should be communicated to healthcare professionals and to the public in an objective and transparent manner [9]. In this paper, we propose a set of minimum standards of evidence required for the evaluation of the scientific validity of genotype-based personalised dietary advice.

## Review

### The context of nutrigenetics

PN should be evaluated in the context of conventional “healthy eating” advice, which forms the basis of public health recommendations aimed at guiding diet and lifestyle habits in populations. These include dietary reference values (DRVs) which aim to promote health by optimising nutrient intake (including setting upper safe limits for nutrients to minimise harm from over-consumption) and to prevent or delay non-communicable diseases ([23, 24, 25]). Although some DRVs are group-specific i.e. they take account of sex, age and physiological state (pregnancy or lactation), they are in essence a “one-size fits all” approach within each group. Consideration of inter-individual variation in requirements is included statistically through setting DRVs. They are designed to meet the needs of 97.5% of the population which means that while the DRV will be greater than the needs of most individuals in the population, the needs of some individuals may not be covered by the established group-based reference values. Recent efforts have refined this approach to include subgroups [25], and a strategy for such refinement was proposed by the EU funded “network of excellence”, EURRECA––Harmonising nutrient recommendations across Europe with special focus on vulnerable groups and consumer understanding ([26, 27]). However, with very few exceptions (e.g. folate), specific methods for evaluation and use of information on genetic variation in the context of general dietary advice are lacking.

Genotype is one class of information [28, 29] that can be used to personalise dietary advice and should be used in combination with other relevant information (e.g. sex, age, anthropometrics, health status, family history or dietary preferences) and never in isolation. In this regard, the appropriate level of evidence for nutrigenetic-based advice is similar to that used in development of conventional nutritional guidelines. However, despite being based on a similar scientific evidence base, such guidelines often differ between countries. This is because, in formulating nutritional guidelines, expert committees make decisions based on the best evidence available, while acknowledging knowledge gaps, and taking into account country-specific issues [25].

## Methodology

A ‘Global Nutrigenetics Knowledge Network’ was established (starting in the Food4Me EU project), involving experts in different areas of PN, to collate all relevant information on genetic variations involved in the nutrient-health relationships and deliver guidelines for the evaluation of evidence for diet-gene interactions. The aims are:

1. To provide a draft framework for assessing the evidence for scientific validity of:
  - Personalised dietary advice based on a specific gene variant
  - Personalised dietary advice based on a specific gene variant that is already available in commercial nutrigenetic tests
2. To create a series of Nutritional GeneCards––based on this draft framework––each of which assesses the evidence for a particular gene-diet/lifestyle interaction.

The Global Nutrigenetics Knowledge Network reviewed guidelines for genetics, medical genetic tests and nutritional recommendations (Evaluation of Genomic Applications in Practice and Prevention (EGAPP), Strengthening the Reporting of Genetic Association Studies (STREGA), Grading of Recommendations Assessment, Development and Evaluation (GRADE), European Food Safety Authority (EFSA) [30–34]) and concluded that these did not fully cover the needs for assessing the evidence for genetics-based personalised dietary advice. EGAPP assesses the clinical utility of a gene, or genes, in conjunction with their variants as predictors of disease, and EFSA assesses the evidence for the potential nutritional or health benefits of specific foods or food components. However, none of these guidelines addresses the evidence from studies that investigate the combined effect of genotype plus diet on health outcomes, which is essential for establishing evidence-based nutrigenetic advice.

The Clinical Pharmacogenetics Implementation Consortium (CPIC) guidelines for pharmacogenetics (gene-drug interactions) have some relevance to nutrigenetics, and we agree with their aim ‘to provide guidance to clinicians as to how available genetic test results should be interpreted to ultimately improve drug therapy, rather than to provide guidance as to whether a genetic test should or should not be ordered’. [35, 36] The CPIC evidence assessment is simpler, relative to nutrigenetics, because (usually) the drug is metabolised by the gene product and has effects over a short time. On the other hand, the risk/benefits equation is such that the evidence would need a higher standard than that applied to nutrition and nutrigenetics.

The ACCE model (Analytical and Clinical Validity, Clinical Utility and Ethics, [37]) for evaluating genetic tests in general was considered an appropriate starting point for genotypic assessment but, in the present context, required modification to recognise that genotypic information will be used in developing PN advice for the public rather than for medical diagnostics. According to ACCE, a medical genetic test should fulfil requirements regarding:

i. Analytical validity––a measure of the accuracy of the genotyping.
ii. Scientific validity––concerns the strength of the evidence linking a genetic variant with a specific outcome
iii. Clinical utility––the measure of the likelihood that the recommended advice or therapy will lead to a beneficial outcome beyond the current state of the art.
iv. Ethical, legal and social implications that may arise in the context of using the test.

The regulation for analytical validity is relatively straightforward, and many countries have laboratory accreditation procedures that cover accuracy and reproducibility [38]. Ethical and legal aspects of nutrigenetics are discussed elsewhere [7, 8]. Clinical utility has strict criteria in the medical sense, demanding strong evidence that a given therapy ‘will lead to an improved health outcome’ [39]. A likelihood ratio (LR) of six or greater is usually considered to be indicative of clinical utility [40]. LR is the likelihood that a given test result would be expected in a patient with the target disorder compared to the likelihood that that same result would be expected in a patient without the target disorder [41]. It could, in principle, be useful to measure the likelihood ratio of the efficacy of nutrigenetic advice relative to generic advice, and a rank of LRs might then be used as an objective measure of the utility of each nutrigenetic test. A caveat is that defining an ‘improved health outcome’ due to nutrigenetic advice in a generally healthy person is very hard to do. Therefore, the present document will concentrate on the development of a draft framework for the assessment of scientific validity, and it will not directly address claims about clinical utility or ‘personal benefit’, see Table 1.

**Table 1:** Why clinical utility is not part of this framework

This framework stops at the assessment of scientific validity. The recommendations provide clear and sufficient detail so that any opinion on health (or clinical) utility can be derived by the user (including the individual, dietician/nutritionist/medical doctor, companies and claim regulation bodies).
Clinical utility is the measure of the likelihood that the recommended therapy or intervention will lead to a beneficial outcome. Clinical utility is the most controversial aspect: it is often difficult to define and must take into consideration many factors including positive or negative psychological or motivational effects on the end user [81]. Others contend that clinical utility can only be thoroughly established through randomised clinical trials (RCT), but these are challenging for the personal genetics environment, includes diet, lifestyle and behavioural changes and has small cumulative effects over decades (see [82, 83] for an example of the current debate). A further problem is the precise definition of a clinical benefit. A gene-diet interaction may not be associated directly with disease risk, such as cardiovascular disease, but with intermediate phenotypes, e.g. lipid levels, hypertension and homocysteine, which are independent risk factors for disease. Some commentators require that clinical utility is demonstrated as a reduction in disease incidence. The majority view accepts that lowering of intermediate risk factors is acceptable (as is the case for phytosterols and their cholesterol lowering properties [50, 84]).
*RCTs* In personalised nutrition research, RCTs with disease incidence as the endpoint are not practically feasible as they will require long-lasting nutritional changes, making compliance difficult and very expensive, at least in terms of *primary* prevention in *healthy* people––apart from any ethical problems. RCTs that address disease incidence reduction in middle-aged or elderly high-risk subjects, secondary prevention in individuals with disease and/or on effects on intermediate biomarkers or risk factors can be useful, but care is required in drawing conclusions. RCTs in nutrition and genetics are often complex, difficult to design and challenging to conduct in a reasonable time frame. Some examples given below illustrate this and may be helpful when interpreting RCT data for personalised diet and lifestyle evidence advice*.*
*Primary prevention in high-risk groups* Genetics × diet × T2DM (type 2 diabetes mellitus)––The T allele of the *TCF7L2* rs7903146SNP has been associated repeatedly with an increased risk of T2DM (2-fold in homozygotes [85]). Compared with non-risk allele carriers, individuals who carry the risk allele and who are at high risk phenotypically (glucose intolerance, pre-diabetes diagnosis) require a longer lasting and a more intense dietary and lifestyle recommendation to divert the trajectory from disease over a period of 12 months and to maintain health gains over a 4-year period [86]. Although useful, these findings have been obtained in clinical trials of *unhealthy* people, who typically were older. Thus, to be precise, it does *not* demonstrate, and cannot be used to claim, that specific dietary modifications in younger, healthier people will prevent the development of glucose intolerance or T2D in those carrying the risk allele. However, this evidence of gene × diet interactions in pre-diabetics is consistent with the evidence from other types of studies in healthy subjects (epidemiological, cohort, effects on biomarkers) and can provide supporting evidence, but not conclusive evidence, that specific dietary guidelines would be appropriate for healthy carriers of this *TCF7L2* risk allele. This example shows how difficult it is to validate a gene-diet interaction but suggests that adjusting the environment will improve the individuals’ health. The same *TCF7L2* genetic variant was assessed in the recently published study from the PREDIMED project [14], a large randomised trial in 7018 high-cardiovascular-risk individuals comparing two Mediterranean (Med) diets and a control diet. *TCF7L2* TT homozygotes at SNP rs7903146 had higher blood glucose levels, total cholesterol, LDL cholesterol and triglycerides but only when adherence to the Med diet was low. Furthermore, incidence of stroke was almost three times higher in TT homozygotes as in the control group, but this increased risk was completely dissolved in the Med diet group (Hazard Ratio, HR = 0.96). Thus, compared to the control diet, both Med diets were effective at reducing both risk biomarkers and disease incidence itself in a genotype specific manner. While this is a strong endorsement of the Mediterranean diet, it is also relevant that the age range was 55 to 80 years. This RCT supports the epidemiological evidence for health benefits of the Med diets for older persons, and those at increased risk of CVD, and can only suggest such benefits for other age groups who carry the *TCF7L2* TT genotype at rs7903146.
*Secondary prevention in subjects with pathology* *MTHFR* × folate × homocysteine on CVD risk––results of several large homocysteine-lowering clinical trials have been published over the last decade, and none reported any benefit in prevention of secondary CVD by folate supplementation. These results have been used widely to declare that there is no evidence that elevated plasma homocysteine levels are relevant for CVD and that there is no benefit in homocysteine-lowering in *primary* prevention [87–89]. However, these were all short-term trials in older people already suffering from (mainly) CVD and taking several medications, where incidence of *further* cardiovascular events was measured. None of the trials were performed in healthy people. Thus, the conclusion from these studies states that over the trial periods there was no apparent benefit in lowering homocysteine in ill people, i.e. as in secondary prevention. However, still lowering homocysteine by using folate may reduce risks of CVD in healthy people with high risk [53, 90, 91]. For instance, the China Stroke Primary Prevention Trial [13, 92], reported on a total of 20,702 adults with hypertension without history of stroke or myocardial infarction who participated in the study. That study compared a single-pill combination containing 10 mg of enalapril and 0.8 mg of folic acid with a tablet containing 10 mg of enalapril only. Among adults with hypertension, the combined use of enalapril and folic acid, compared with enalapril alone, significantly reduced the risk of first stroke (HR = 0.79). Analysis of the *MTHFR* 677 genotype showed further that the TT genotype had the largest risk reduction in the highest folate quartile (HR = 0.24), suggesting that individuals with the TT genotype may have a greater folate requirement. *MTHFR* × *riboflavin* × *hypertension*––several RCTs have demonstrated that riboflavin supplementation contributes to blood pressure-lowering specifically in hypertensive carriers of the 677T allele [52, 93–95]. The trials do not prove primary prevention (i.e. they do not demonstrate that increasing riboflavin in 677T normotensives prevents development of hypertension), and they do not prove the ultimate health benefit of riboflavin to reduce incidence of heart disease. However, reducing blood pressure is considered to be a health benefit in itself, and although the results cannot be used to establish a genotype specific role of riboflavin in *primary* prevention of hypertension, they can be used to support other types of studies. Overall, outcomes of RCTs can be useful for nutrition/lifestyle advice, but they need to be interpreted with care. Furthermore, it must be accepted that conducting an RCT in *young healthy people* with the aim of investigating the effect of nutrition on actual reduction of *disease incidence* over the long term is not feasible either ethically, economically or scientifically (see also [96] for discussion). On the other hand, the use of RCTs to study the effects on biomarkers that quantify *health* (i.e. not simple risk markers of impending disease) is a promising new approach [97].

## Proposed framework for scientific evidence assessment

The components relevant to this assessment are summarised in Table 2. This draft framework will be used in ‘nutrition gene cards’ that assess the evidence supporting specific gene × diet interactions and its relation to a specific health outcome (see below). In the framework, we implement components used in systematic reviews [42], including the development of the search strategy and the assessment of the study quality.

**Table 2:** Framework for stepwise assessment of the evidence relating to gene × diet interactions

Scientific validity assessment criteria	
Study quality rating (A, B, C, D): * Interventional or observational design * Prospective and retrospective approach * Randomised, placebo controlled and blinded * Study power (high subject number with ‘effect’ allele) * Effect magnitude * *P* values, false discover rate (FDR) and multiple testing * Replication study in different populations and meta-analysis Type of gene × diet Interaction: * Direct phenotype * Intermediate phenotype * Indirect phenotype Nature of the genetic variant * Causal * In LD with functional variant * Associated but unknown function Biological plausibility * Rated as high/medium/low/unknown Scientific validity score for gene × diet interaction * Convincing * Probable * Possible * Not demonstrated	

*P values must be at least .05 to be significant. The *P* value must remain within .05 after correcting for multiple testing, e.g. Bonferroni

^a^The ‘effect magnitude’ required depends on the type of study. For example, the effect of folate on high homocysteine in carriers of the effect allele in *MTHFR* should be a return to normal within a few weeks of starting the intervention. The magnitude of reduction of blood pressure would be acceptable for as little as 1 mmHg, and any risk reduction, however small, for cardiovascular disease would be adequate

Scientific validity may be graded as (the percentages are based on the Guidance on Uncertainty in EFSA Scientific Assessment [43]):

**Table:** 

Probability term	Subjective probability range (%)
A. Convincing	> 90
B. Probable	66–90
C. Possible	33–66
D. Insufficient	< 33

“Convincing” should be based on several (at least three) strong studies with high subject numbers, showing the relation and/or mechanistic knowledge, “probable” based on several studies showing the relation and/or some mechanistic understanding and “possible” based on a few studies showing the relation. See below in the text for a fuller explanation.

Scientific validation determines the strength of the evidence for an interaction between a specific genetic marker or set of markers and a dietary component or pattern on a health outcome of interest (such as disease or risk factors for disease). The scientific validity criteria for genetic-based dietary advice within this proposed framework include (i) study design and quality and (ii) biological mechanism including the nature of the genetic variant(s) and biological plausibility, as discussed below. The probability term is the overall judgement of the evidence provided, and in this sense ‘it is possible for an evidentiary conclusion based on many papers, each of which may be relatively weak, to be graded as ‘moderate’ [probable] or even ‘strong’ [convincing], if there are multiple small case reports or studies that are all supportive with no contradictory studies’ [36].

1. Study design and quality

All studies reporting on genetic interactions should adhere to the STREGA guidelines [33]. STREGA was developed with aim of improving the transparency of reporting involving ‘population stratification, genotyping errors, modelling haplotype variation, Hardy-Weinberg equilibrium, replication, selection of participants, rationale for choice of genes and variants, treatment effects in studying quantitative traits, statistical methods, relatedness, reporting of descriptive and outcome data, and the volume of data issues that are important to consider in genetic association studies’ [33].

Only studies that include STREGA guidelines should be considered when assessing a gene-diet interaction. In addition to the STREGA guidelines, the intake of the dietary component of interest should be reported quantitatively; for the description on the quantification of diet intake, see [44].

2. Biological plausibility

*‘*a gene–environment interaction will only be accepted if it can be reproduced in two or more studies and also seems plausible at the biological level.*’* [45]

Biological plausibility is a judgement based on the collected evidence of a gene × diet interaction on a phenotype. For example, smoking introduces carcinogens into the body, which could cause DNA damage, increased mutational events, and consequently increasing risk of cancer [46]. Thus, a validated gene × nutrition interaction such as *GSTM1* × cruciferous vegetable consumption leading to reduction of DNA damage ([47, 48]) is consistent with an association between *GSTM1* × cruciferous and reduced cancer risk ([49]).

Gene × diet interactions, the scientific underpinning of this paper, are defined here as the particular physiological response to a dietary component which occurs only––or is more pronounced––in persons with a specific version of a gene (or genes). For example, a certain genotype may be associated with increased concentrations of LDL cholesterol and increased risk of cardiovascular complications, but only in the case of long-term, higher-than-average saturated fat consumption [28, 50]. Evidence of this type of interaction may be used to develop an appropriate dietary recommendation (e.g. consuming lower than average intake of saturated fats) which may reduce or eliminate the potential negative consequences associated with the specific genotype and may be targeted to specific groups of people.

(i) Gene X diet interaction

We identify three broad types of gene × diet interactions: direct, intermediate and complex.

- A ‘direct’ interaction is a mechanistic interaction between the genetic variant and the dietary component on the health biomarker. This type of interactions is most similar to metabolism of a drug by the gene encoding the enzyme that directly metabolises the drug.
- An intermediate interaction is a mechanistic interaction between the genetic variant and the dietary component on the health biomarker, but other processes also affect the level of the biomarker.
- An indirect interaction is the case where a mechanistic interaction between the genetic variant and the dietary component on a health biomarker, including disease, is affected to some extent by the gene × diet interaction but is also influenced by many other, possibly unknown processes, and it may take years for symptoms to manifest. This type of interaction may not be fully explained physiologically or may be only demonstrated statistically.

A well-researched example is the case of supplementation with folate (substrate) and/or riboflavin (cofactor) in persons who are carriers or non-carriers of the methylenetetrahydrofolate reductase (*MTHFR)* C677T polymorphism (rs1801133) where ‘T’ is the allele associated with reduced enzymatic activity. Examples with the folate-related enzyme MTHFR are as follows:

- Direct: e.g. *MTHFR* × folate → levels of homocysteine [51]
- Intermediate: e.g. *MTHFR* × riboflavin → blood pressure [52]
- Indirect: e.g. *MTHFR* × folate /riboflavin → cardiovascular disease [53]

(ii) Nature of genetic variant

This draft framework considers three major classes of genetic variants, with differing strengths of evidence.

- The genetic variant has a demonstrated causal effect on the function of the gene product, e.g., on enzyme activity or protein abundance, which provides a biologically plausible explanation for the gene-diet interaction.
- The genotyped variant may not itself affect the protein of interest but it may be in linkage disequilibrium (LD) with another relevant functional variant––one SNP is said to ‘tag’ the other [54, 55]. Evidence would be required to validate both the LD score and the putative gene × diet interaction. In addition, the results also would be applicable only to the population(s) in which high LD has been established.
- The effect of the SNP on function of the gene product is unknown and is based only on a statistical association for the gene × diet effect [54, 55].

Examples of these three types of genetic variants are:

- *Causative*: SNP rs1801133 (TT) reduces the activity of the MTHFR enzyme [56]
- *In LD with known causative variant:* the SNP rs4341, which is in LD with an InDel variant (rs4646994) in the same gene, *ACE*, that affects the plasma angiotensin converting enzyme (ACE) levels ([57, 58])
- *Unknown*: e.g. rs7903146 (CT and TT) intron SNP in the *TCF7L2* gene is linked to type 2 diabetes risk. An interaction with carbohydrate and diet has been demonstrated, but the effect of this polymorphism, if any, on function of the corresponding gene product has not been characterised. This SNP may be in LD with a functional SNP or has an as yet uncharacterised function ([59, 60]).

### Scientific validity assessment of a putative gene × diet interaction

Assessing the validity of a putative gene × diet interaction is generally complex, and as knowledge deepens in the area of nutrigenetics, assessment of its validity will develop. We propose a pragmatic way to assess the validity by relying initially on semi-quantitative measures that can be improved with additional data. The numbers used below to assess the validity should be considered an arbitrary but coherent guideline based on suggested power calculations, and precision of measurements of diet exposure and outcome [45, 61, 62], plus experience based on successfully repeated gene × diet studies published to date.

The assessment should explicitly specify for which subgroup (e.g. sex, ancestral background and other relevant subgroups) the evidence is collected.

*Convincing*

- Two independent studies that have shown the relationship between the gene-diet interaction and the specific health outcome. Together those studies should include at least 100 subjects carrying the effect allele (i.e. the presumed functional variant or in LD with the functional variant) for intervention studies and at least 500 subjects for observational studies. Specifically, if the frequency of the effect allele is 10% that means that a total of at least 1000 and 5000 subjects for intervention and observational studies, respectively.
- Biological mechanism fully understood or largely explained.
- Biological mechanism partly explained and having one correlative study that at least includes 50 (intervention) or 250 (observational) subjects carrying the effect allele.

*Probable*

- Two independent studies that have shown the correlation between the gene-diet and health outcome (together < 100 (intervention) or 500 (observational) subjects carrying the effect allele)
- One study that has shown the correlation between the gene-diet and health outcome including at least 100 (intervention) or 500 (observational) subjects carrying the effect allele.
- Biological mechanism partly explained and having one small correlative study (< 50 (intervention) or 250 (observational) subjects carrying the effect allele)

*Possible*

- One study has shown the correlation between the gene-diet and health outcome (< 100 (intervention) or 500 (observational) subjects carrying the effect allele)
- Biological mechanism partly explained and having one small correlative study (< 50 (intervention) or 250 (observational) subjects carrying the effect allele)

*Not demonstrated*

- Any other studies, excluding the abovementioned studies

Based on the factors described above, an overall assessment of all evidence can be made to arrive at a combined score on the scientific validity, and our degree of confidence in that assessment, of the gene × diet interaction being predictive of the outcome of interest. Some examples include:

- *Convincing––very high confidence. MTHFR* and homocysteine concentrations which are influenced by dietary folate: a large numbers of studies including randomised trials, very consistent results, direct effect of the genetic variation on enzyme activity, and high biological plausibility [63, 64].
- *Probable––high confidence. SOD2* × antioxidants and prostate and breast cancer risk have been demonstrated consistently in gene × diets studies. Some large studies show a reduction in cancer risk when antioxidant intake is high. The biological plausibility is high, but all available evidence comes from prospective or retrospective observation trials and not randomised trials [65–67].
- *Possible––BCMO1* × carotenoid and retinal levels. The *BCMO1* gene product is an enzyme that converts β-carotene to vitamin A (retinal). Certain *BCMO1* alleles are associated with higher plasma β-carotene levels, and such allele variants may result in lower enzyme activity. However, there is no clear demonstration on the effect of dietary advice [68, 69].
- *Not demonstrated. FADS2* × breastfeeding and IQ––three published studies, with three conflicting results:

- ○ In 3269 children, in two cohorts (Britain and New Zealand), an increase in IQ but only in breastfed infants who were carriers of the C-allele for the FADS2 rs174575 SNP which is the major allele intronic tag SNP associated with higher docosahexaenoic acid (DHA) [70].
- ○ Second study examined 5934 British children––breastfed children with the GG genotype were actually associated with higher IQ [71].
- ○ Third study of 1431 Australian children––there were no differences in IQ either for breastfeeding or genotype at the FADS2 rs174575 SNP [72].

## Conclusion

We have developed a draft framework of criteria allowing for the assessment of the quality of the evidence for PN advice based on individual gene variants. This framework is intended to establish the scientific validity of reported gene(s) × diet interactions and to determine the likelihood that the predicted outcomes will be consistent and reproducible. The fundamental requirement of a nutrigenetic test, as with any health-related test, is that the results should clearly indicate a diet-related recommendation that is beneficial in relation to a concrete aspect of health or performance. Any such advice should fulfil all requirements set out in the framework described here. We have not addressed the ethical aspects of nutrigenetic testing and whether professional pre- and post-test counselling is required because this has been addressed and discussed elsewhere ([8, 11, 15–18]). The framework described here is limited to evidence of validity, without which advice should not be given.

The rapidly developing commercial environment, in conjunction with the interest of public health bodies in PN, has created a strong need for formal assessments of the evidence. A large number of gene × diet interactions which might affect phenotypes pertinent to commonly occurring diseases have been reported and this number will increase as data become available from genome-wide GxE (Gene–environment interaction) studies ([73, 74]). Furthermore, it is likely that only a proportion of these reported gene × diet interactions currently could be judged as valid by the criteria described here and therefore have a potential benefit in PN.

The nutrigenetics research community organised in the ‘global personalised nutrigenetics knowledge network’ can contribute to the translation of the accumulating knowledge into practice by using these proposed guidelines. Regular reviews (e.g. on a bi-annual basis) of current knowledge of the most studied polymorphisms may generate additional, scientifically valid associations and hence provide recommendations about which polymorphisms may inform genotype-based dietary advice. We will also include reviews of genetic variants that do not meet these standards. The practical output for this exercise will be the publication of nutrient-gene cards in this journal Table 3.

**Table 3:** Application of the framework

This framework described here can be used by dietitians, nutritionists, doctors and genetic counsellors (and customers too) to judge the soundness of gene(s)-diet interactions. As such, nutrigenetics may develop to be part of standard of care. Ultimately, the use of evidence-based nutrigenetic tools should be the basis for dietary advice aimed not only to reduce the risk of disease but also as a tool to optimise diet to promote long-term health.
*Nutrition gene cards* This framework can be used to assess the specific relation between a gene (genes) and nutrient for publication. For this purpose, the Global Nutrigenetics Knowledge Network also developed the concept of a nutrition gene card, which is a short publication using this framework and assessing a specific gene-diet interaction. These short publications should include all the aspects of Table 2.
The nutrition gene cards should adhere to some basic rules: 1. Studies should be identified in a systematic way, and studies that contain proven statistical flaws will be explicitly removed from consideration. 2. The nutrition gene card should be peer reviewed. 3. All guidelines should be publicly available as an online educational resource. 4. All researchers are invited to contribute to the nutrition gene card––either to update current cards or to propose new gene-diet cards. Presumably, the lessons learned by writing these nutrition gene cards will sharpen the framework as described in this paper.

Adoption of nutrigenetics and genetic testing has been slow in the healthcare system, and we hope that these guidelines accelerate informative genetic testing for education and practice. In a recent survey of 373 Canadian registered dietitians, 76% responded that they do not have sufficient knowledge of nutrigenetics. In spite of this, the majority stated ‘that genetic testing and their results have poor accuracy, and that there is a lack of scientific evidence’ [75]. These contradictory responses suggest that there is a lack of impartial objective material available for educating dietitians, nutritionists and other healthcare professionals ([75]). However, the American Dietetic Association recently has published a position paper on nutritional genomics [76]. One important part of that paper interprets nutrigenetics differently by stating that ‘The practical application of nutritional genomics for complex chronic disease is an emerging science and the use of nutrigenetic testing to provide dietary advice is not ready for routine dietetics practice as most chronic diseases, such as CVD, diabetes, and cancer are multigenetic and multifactorial and therefore genetic mutations are only partially predictive of disease risk’. We, however, state that the primary goal of nutrigenetics in the context of this paper is not to predict risk but to develop genotype-based (one or more gene) dietary advice supplementing the standard guidelines for everyday use in the framework of a health-promoting nutrition.

A genetic test for disease risk prediction would require different levels of evidence since disease risk predictions require the contribution of many SNPs. Determining risks relies on SNPs identified from genome-wide association studies (GWAS), many of which are in introns or gene deserts and have unknown functions. Since all GWAS and most nutrigenetic testing is based on (study) population averages, converting population attributable risk to personal risk is not possible [77]. A risk prediction could involve gene × diet interactions (i.e. nutrigenetics), but nutrigenetics would not be the primary source of the risk prediction. For example, MyGeneRank is a mobile app that uses 50+ GWAS SNPs, mostly of unknown function, in a genetic risk score (GRS) to predict coronary artery disease risk. In a second part of the test, it could be useful to use nutrigenetics (using SNPs and other variants, like InDels) to help with improving diet, which in turn can reduce the actual risk compared to the GRS risk value [78].

In the commercial environment, the field of personal genetics and nutrigenetics are under increasing scrutiny from regulators. For example, the US Food and Drug Administration (FDA) and the Federal Trade Commission (FTC) limited the scope of direct to consumer products and services ([79]). Many companies would likely prefer to commercialise reputable, useful products and services, but the absence of clear regulations or guidelines mitigates against such developments. The lack of educational resources and the variable quality of tests currently on the market limits the ability to translate research to utility and reinforces the need for a framework for assessing validity of gene × diet interactions. We have studied and incorporated recommendations and guidelines from expert groups in medical genetics and other types of medical tests (e.g. EGAPP and GRADE [30–32]), with some modification for the specific and different circumstances and requirements of nutrition.

Our approach has some limitations and strengths. One limitation is the general heterogeneity and mixed quality of studies combining both genetic and nutritional analysis, often making it difficult to compare apparently similar studies analysing the same genetic variants and dietary components. Nutrition research in general is prone to inconsistency because of the high complexity and the subtle effects of nutrition on long-term health, the difficulties in accurate dietary assessment, food-food or nutrient-nutrient interactions, environmental context which can alter nutrient hosts interactions and compliance to a specific diet. These same issues also restrict the use of findings from meta-analysis. Inevitably, any assessment of nutrition and nutrigenetics can be only semi-quantitative at best. We consider that our approach has the benefit of creating a formal and generic model for the assessment of such evidence and will guide more focussed debates on specific points, which may be judged in different ways. Moreover, the framework and associated resources will allow stakeholders such as dieticians, nutritionists and genetic counsellors to improve their knowledge of nutrigenetics and at the same time will provide a resource to assess the various tests that are offered. This framework may encourage a greater standardisation of research protocols, supporting other initiatives such as PhenX [80], as well as the reporting of novel and replicated gene-environment interactions in other populations.

## Abbreviations

ACCE: Analytical and Clinical Validity Clinical Utility and Ethics
ACE: Angiotensin I converting enzyme
BCMO1: Beta-carotene 15,15′-monooxygenase 1
CVD: Cardiovascular disease
DRV: Dietary reference values
DTC: Direct to consumer
EFSA: European Food Safety Authority
EGAPP: Evaluation of Genomic Applications in Practice and Prevention
FADS2: Fatty acid desaturase 2
FDA: Food and Drug Administration
FTC: Federal Trade Commission
GRADE: Grading of Recommendations Assessment, Development and Evaluation
GxE: Gene–environment interaction
LD: Linkage disequilibrium
LR: Likelihood ratio
MTHFR: Methylenetetrahydrofolate Reductase
PN: Personalised nutrition
SOD2: Superoxide dismutase 2
STREGA: Strengthening the Reporting of Genetic Association Studies
T2D: Type 2 diabetes
TCF7L2: Transcription factor 7-like 2
